# *In situ* neutron diffraction to investigate the solid-state synthesis of Ni-rich cathode materials

**DOI:** 10.1107/S1600576723004909

**Published:** 2023-06-23

**Authors:** Damian Goonetilleke, Emmanuelle Suard, Benjamin Bergner, Jürgen Janek, Torsten Brezesinski, Matteo Bianchini

**Affiliations:** a Battery and Electrochemistry Laboratory (BELLA), Institute of Nanotechnology, Karlsruhe Institute of Technology (KIT), Hermann-von-Helmholtz-Platz 1, 76344 Eggenstein-Leopoldshafen, Germany; b Institut Laue–Langevin (ILL), BP 156, 71 Avenue des Martyrs, 38042 Grenoble, France; c BASF SE, Carl-Bosch-Strasse 38, 67056 Ludwigshafen, Germany; d Institute of Physical Chemistry and Center for Materials Research (ZfM/LaMa), Justus-Liebig-University Giessen, Heinrich-Buff-Ring 17, 35392 Giessen, Germany; e University of Bayreuth, Bavarian Center for Battery Technology (BayBatt), Universitätsstrasse 30, 95447 Bayreuth, Germany; Uppsala University, Sweden; The European Extreme Light Infrastucture, Czech Republic

**Keywords:** *in situ* neutron diffraction, synthesis, cathodes, Ni rich, solid state, high annealing temperatures, Rietveld refinement, lithiation

## Abstract

Studying chemical reactions in real time can provide unparalleled insight into the evolution of intermediate species and can provide guidance to optimize the reaction conditions. For solid-state synthesis reactions, powder diffraction has been demonstrated as an effective tool for resolving the structural evolution taking place upon heating. The synthesis of layered Ni-rich transition-metal oxides at a large scale (grams to kilograms) is highly relevant as these materials are commonly employed as cathodes for Li-ion batteries. In this work, *in situ* neutron diffraction was used to monitor the reaction mechanism during the high-temperature synthesis of Ni-rich cathode materials with a varying ratio of Ni:Mn from industrially relevant hydroxide precursors. Rietveld refinement was further used to model the observed phase evolution during synthesis and compare the behaviour of the materials as a function of temperature. The results presented herein confirm the suitability of *in situ* neutron diffraction to investigate the synthesis of batches of several grams of electrode materials with well-controlled stoichiometry. Furthermore, monitoring the structural evolution of the mixtures with varying Ni:Mn content in real time reveals a delayed onset of li­thia­tion as the Mn content is increased, necessitating the use of higher annealing temperatures to achieve layering.

## Abstract 2

*In situ* neutron diffraction is demonstrated as a suitable technique to track phase evolution during synthesis of layered oxide compounds. The synthesis of a series of cathode materials for Li-ion batteries with different Ni/Mn ratio is investigated, in batches comparable to standard laboratory conditions, demonstrating the delayed onset of lithiation with increasing Mn content.

## 1. Introduction

The ability to observe chemical processes in real time offers unparalleled insight into the phase evolution, kinetics and behaviour of product materials while the reactants are not under equilibrium conditions. For solid-state reactions, the use of X-ray or neutron scattering techniques has proven fruitful for monitoring such processes (Bianchini, Wang *et al.*, 2020 ▸; Bremholm *et al.*, 2008 ▸; Jensen *et al.*, 2014 ▸; Rao *et al.*, 2015 ▸). Solid-state reactions are always driven by a thermodynamic driving force (Δ_r_ *G*), typically influenced by temperature or partial pressure; however, often kinetic aspects also play an important role in stabilizing metastable phases, which can in turn prevent the formation of the thermodynamically stable phases (Shoemaker *et al.*, 2014 ▸; Jiang *et al.*, 2017 ▸; Martinolich & Neilson, 2017 ▸). Understanding the processes taking place during these reactions has remained a challenge within solid-state chemistry, with reactions typically taking place under harsh conditions including high temperature or pressure, and under controlled atmospheres (Kohlmann, 2019 ▸). For this reason, *in situ* synthesis investigations are gaining increased attention. In tandem with the improved accessibility of advanced characterization techniques, beamlines at both synchrotron and neutron sources now offer specialized sample environments that can be used to study materials under non-ambient conditions, such as applied temperature, pressure or even magnetic fields (Hansen & Kohlmann, 2014 ▸; Goonetil­leke & Sharma, 2019 ▸; Bianchini *et al.*, 2022 ▸; Bailey, 2003 ▸; Salamat *et al.*, 2014 ▸). Variable-temperature sample environments offer the ability to probe materials during both heating and cooling above or below ambient temperatures. High-temperature sample environments in particular have made possible novel studies that can be used to resolve the phase evolution and pathways of solid-state reactions, including gas–solid or liquid–solid reactions (Møller *et al.*, 2014 ▸; Hills-Kimball *et al.*, 2017 ▸; Herzog *et al.*, 1996 ▸; Günter *et al.*, 2001 ▸; Shen *et al.*, 2006 ▸), for a variety of different materials, including metal hydrides for application in hydrogen storage (Polanski *et al.*, 2010 ▸; Norek *et al.*, 2011 ▸; Sato *et al.*, 2013 ▸), metal nitrides that may act as semiconductors or express ferromagnetism (Choi *et al.*, 1994 ▸; Brendt *et al.*, 2009 ▸), metal oxide or carbide catalysts (Papavasiliou *et al.*, 2006 ▸; Schnepp *et al.*, 2015 ▸), and intermetallic alloys with unique physical and mechanical properties (Novák *et al.*, 2013 ▸; Xiong *et al.*, 2019 ▸; Walsh & Freedman, 2018 ▸). Such studies are not only exemplary for understanding material synthesis but also highly pertinent for resolving the mechanisms behind processes used in the chemical industry for large-scale production of functional materials.

Among these, one important family of functional materials are those used as rechargeable battery materials, in particular, layered transition metal (TM) oxides, which have enabled the success of lithium-ion batteries (Manthiram, 2020 ▸; Grey & Hall, 2020 ▸). These devices have high specific energy and power with respect to both volume and weight compared with other secondary energy-storage systems (Delmas *et al.*, 2021 ▸). The layered structure allows for the reversible intercalation of lithium as the battery is charged and discharged. For high specific energy applications, such as electric vehicles, compounds with compositions LiNi_1−*x*−*y*_Mn*_x_* Co*_y_* O_2_ and LiNi_1−*x*−*y*_Co*_x_* Al*_y_* O_2_ have been widely adopted (Zubi *et al.*, 2018 ▸; Reinhardt *et al.*, 2019 ▸). Recently, both academia and industry have focused on increasing the nickel content in these materials with the intent of improving specific energy by taking advantage of the high redox activity of nickel (Muralidharan, Essehli, Hermann, Amin *et al.*, 2020 ▸; Muralidharan, Essehli, Hermann, Parejiya *et al.*, 2020 ▸; Yang *et al.*, 2021 ▸; Hebert & McCalla, 2021 ▸). Meanwhile, issues with the expense and sourcing of cobalt, due to its toxicity and scarcity, have also triggered the development of phases with minimized cobalt content (Ryu *et al.*, 2021 ▸; Luo *et al.*, 2022 ▸; Liu *et al.*, 2021 ▸). However, preparing these materials via solid-state synthesis, which is desirable for scalability, presents difficulties due to their structural instability at the desired calcination temperatures (Kurzhals *et al.*, 2022 ▸), especially as compositions approach the LiNiO_2_ end member (Bianchini, Fauth *et al.*, 2020 ▸; Bianchini *et al.*, 2019 ▸; Nitta *et al.*, 1995 ▸). In particular, precisely controlling the resulting stoichiometry of the synthesized material at temperatures close to 700 °C in an O_2_ gas flow is challenging, and even subtle variations can have a significant influence on the structure, morphology and ultimately electrochemical performance of the synthesized material (Kurzhals *et al.*, 2021 ▸; Riewald *et al.*, 2022 ▸; Mesnier & Manthiram, 2020 ▸). Increasing the manganese content at the expense of cobalt and nickel has been explored as a route towards producing economical and high-capacity cathode materials, with the side effect of also necessitating higher synthesis temperatures and improving atmospheric stability (Wang *et al.*, 2022 ▸; Hu *et al.*, 2018 ▸; Yin *et al.*, 2020 ▸; Pimenta *et al.*, 2017 ▸).

**Figure 1:**
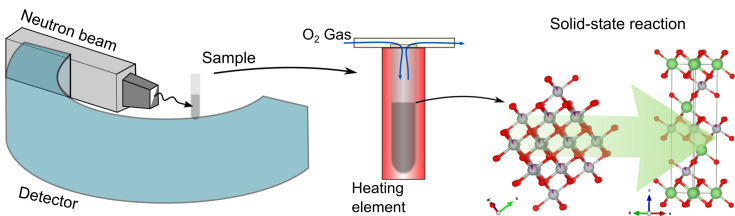
A schematic illustration of a setup for *in situ* neutron diffraction to investigate solid-state synthesis reaction at elevated temperatures. The crystal structures on the right refer to cubic Ni_0.9_Mn_0.1_O transforming into layered LiNi_0.9_Mn_0.1_O_2_ during concurrent li­thia­tion and oxidation.

The synthesis and decomposition of LiNiO_2_ have previously been detailed in real time using variable-temperature *in situ* synchrotron X-ray diffraction, which provides excellent angular and time resolution (Bianchini, Fauth *et al.*, 2020 ▸). However, the nature of the synchrotron X-ray beam limits the volume and mass of the sample that can be probed to a thin capillary (<1 mm diameter) and just several milligrams of material. In more realistic laboratory conditions, materials are typically prepared at the gram scale, in larger alumina crucibles. Such conditions can be approached using neutron powder diffraction. Due to the high penetration depth of neutrons in most materials, neutron powder diffraction offers the possibility to probe a much larger amount of sample, with a transmission distance of ≈1–2 cm diameter allowing a mass loading of several grams (Goonetilleke & Sharma, 2019 ▸; Peterson & Kearley, 2015 ▸). This allows scattering from a larger number of grains and thus improves the statistics. Hence, for studying solid-state synthesis reactions in particular there are several advantages: not only is the larger amount of sample more comparable to real synthesis conditions in the laboratory but also the loss of Li precursors through reaction with the quartz capillary can be reduced. This loss of precursors has been observed in previous studies and presents a significant issue, due to the small amount of sample and large contact surface with the tube (Geßwein *et al.*, 2022 ▸; Bianchini, Fauth *et al.*, 2020 ▸). Li_4_SiO_4_ impurities were observed, undermining the targeted stoichiometry of the final LiNiO_2_ product. However, this issue is mitigated using the experimental setup described here for *in situ* neutron diffraction (Fig. 1 ▸), as will be shown in the following. Finally, neutrons are valuable probes for lithium, due to its negative scattering length. This makes it detectable because of the high contrast with the other atoms present in the structure, while this is hardly the case for X-rays due to its low atomic weight (Sears, 1992 ▸; Chantler *et al.*, 2003 ▸). While *in situ* synchrotron X-ray and neutron studies can provide similar information about the intermediate phases that form during synthesis, such as lattice parameters or phase quantification, it is anticipated that neutrons will enable more precise refinement of atomic parameters such as the Li occupancy of the phases, which are important to understand the progression of li­thia­tion in the reactions studied here. The strengths of synchrotron X-ray radiation are in providing very precise lattice parameters, while the high intensity ensures that minor intermediate phases are easily observed (Goonetilleke *et al.*, 2019 ▸; Peterson & Papadakis, 2015 ▸).

In this work, *in situ* neutron diffraction was used to monitor the reaction mechanism during the high-temperature synthesis of the layered cathode material LiNiO_2_, as well as related phases where Ni is partly substituted by Mn: LiNi_0.9_Mn_0.1_O_2_ and LiNi_0.75_Mn_0.25_O_2_. The synthesis was carried out using hydroxide precursors supplied by an industrial research partner, which are also used in the industrial scale production of Ni-rich cathode materials. Rietveld refinement was further used to track the structural evolution of the hydroxide precursors to the final layered phases, and the three materials are qualitatively and quantitatively compared.

## 2. Experimental

Three synthesis reactions are investigated here, targeting LiNiO_2_, LiNi_0.9_Mn_0.1_O_2_ and LiNi_0.75_Mn_0.25_O_2_: hereafter referred to as products LNO, NM9010 and NM7525, respectively. The materials were synthesized from precursors of the appropriate stoichiometry, *i.e.* TM hydroxides, TM(OH)_2_, obtained via coprecipitation (BASF SE). LiOH·H_2_O (BASF SE) was used as the lithium source. A stoichiometric lithium excess of *n*(TM):*n*(Li) = 1:1.01 was used. The total mass of each precursor mixture was ≈11 g. The precursors were mixed using a laboratory blender (Kinematica AG) to ensure homogeneity.

For the *in situ* synthesis experiments, ≈5 g of the blended precursor mixtures were filled into a quartz tube of diameter 10/12 mm (internal/external) to a height of ≈50 mm, targeting a final product mass of ≈3 g. Neutron diffraction patterns were recorded in transmission geometry on the high-intensity diffractometer D20 at the Institut Laue–Langevin (ILL) in Grenoble, France (Hansen *et al.*, 2008 ▸). This instrument is equipped with a 1D position-sensitive detector that allows for continuous detection of diffraction patterns in the range 0 < 2θ < 153.6°. Primary heating and neutron diffraction data collection were conducted in a resistive vanadium vacuum furnace operating under a pressure of 10^−2^ mbar. The sample and the silica tube were placed at the centre of a 20 mm internal diameter V heating element (20–40 mm thick). A boron carbide (B_4_C) mask positioned 3200 mm from the main reactor aperture and 50 mm from the sample focal plane limited the neutron beam height to 42 mm with a divergence of 3 mm. *K*-type thermocouples were positioned 3 mm below the sample and used to control the furnace temperature. The samples were heated under an O_2_ flow to the desired annealing temperature for the target compositions (700, 770 and 900 °C) at a ramp rate of 2 °C min^−1^, and held for at least 2 h before the heating element was turned of and the sample allowed to cool naturally. During heating of the precursor mixtures, patterns were collected with an acquisition time of 5 min in the range 0.029 < 2θ < 149.729°. The incident wavelength of λ = 1.5422 (2) Å was determined by refinement against patterns collected from the same precursor materials at the ALBA synchrotron (Bianchini, Fauth *et al.*, 2020 ▸).

Visualization of the recorded diffraction data was carried out using the *Large Array Manipulation Program* (*LAMP*) (Richard *et al.*, 1996 ▸). Diffraction patterns were analysed using the Rietveld analysis software *GSAS-II* (Toby & Von Dreele, 2013 ▸). Prior to refinement of the patterns, background subtraction was performed using the profile obtained from diffraction patterns collected from an empty quartz tube that was heated to selected temperatures relevant for the *in situ* synthesis reaction, as shown in Fig. S1 of the supporting information. Background subtraction allows the background profile to be fitted using fewer terms, minimizing the number of refined parameters. For sequential refinement of structural models against the *in situ* data, the refined parameters for major phases were typically a zero correction, scale factors, peak-shape parameters and lattice parameters. The initially observed LiOH phase was fitted using a Pawley refinement, and thus does not contribute to the refined weight fractions of the cubic and layered phases discussed below. The cubic rock salt-type phase was modelled using a structural model with space group 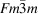 . All the materials synthesized here are isostructural with α-NaFeO_2_, and thus were modelled using a layered structure with space group 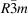 . An example of an input structural model is shown in Table S1 of the supporting information. The site occupancy of the TM site (3*a*) in the structure was adjusted to reflect the composition of the different cathode materials, with the Mn content fixed and Li and Ni occupancies allowed to refine. The Li site (3*b*) was assumed to be fully occupied by Li or Ni, and their relative amount was refined. Due to the strong correlation of atomic displacement parameters (ADPs) and site occupancy factors (SOFs), which are expected to vary dynamically during the synthesis experiments, the ADPs of each site were fixed to established reasonable values previously determined in structural studies of TM-based cathode materials, allowing the site occupancies to be refined freely (Yin *et al.*, 2019 ▸, 2018 ▸).

## 3. Results and discussion

**Figure 2:**
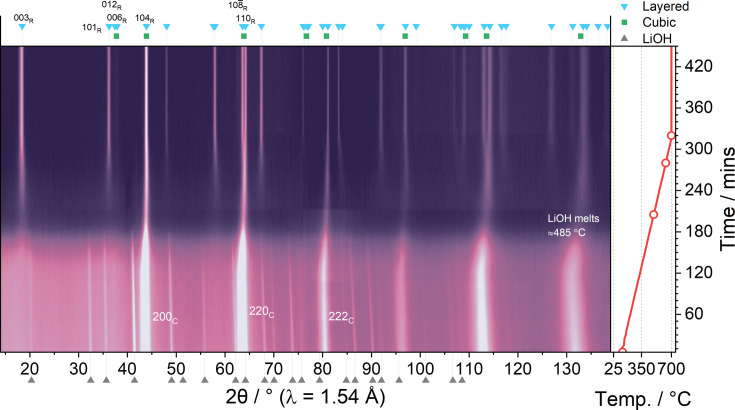
Evolution of diffraction data as a function of temperature during heating of the NiO + LiOH mixture. Labels indicate the positions of key reflections from the cubic (C) and layered (R) phases. The open red circles in the temperature plot indicate the positions of patterns shown in Fig. 3 ▸.

The synthesis of LNO has previously been well described using variable-temperature *in situ* synchrotron X-ray diffraction (Bianchini, Fauth *et al.*, 2020 ▸). To validate the method and compare the behaviour of the same system at a larger scale, the synthesis with analogous precursors is studied here using *in situ* neutron powder diffraction. Fig. 2 ▸ shows a contour plot of the diffraction patterns collected during the heating of precursors NiO and LiOH to form LNO. These were obtained by dehydration of the respective hydroxides via a pre-calcination treatment at 350 °C for 6 h. The reaction mechanism can be considered as taking place in three main regions: region I – the gradual li­thia­tion of the cubic NiO phase to form Li*_y_* Ni_1−*y*_O, where Li is distributed with Ni randomly within the octahedral sites in the structure (*y* > 0 already at the beginning of the *in situ* experiment due to the pre-calcination) (Bianchini, Fauth *et al.*, 2020 ▸); region II – the diffusion of Li^+^ ions within the cubic structure to form a more ordered structure (a ‘defective’ layered phase – Li_1−*z*_Ni_1+*z*_O_2_); and region III – the formation of the fully ordered layered structure LNO towards the end of the synthesis. However, as discussed above, the formation of a perfectly layered structure is unlikely to occur via solid-state synthesis, *i.e.* a small amount of residual Ni is always observed to be present on the Li site (off-stoichiometry *z*, or Li deficiency) (Bianchini *et al.*, 2019 ▸; Croguennec *et al.*, 2009 ▸; Goonetilleke *et al.*, 2023 ▸). In the diffraction data shown in Fig. 2 ▸, broad reflections from the cubic NiO phase can be observed at the beginning of the experiment, in addition to reflections from the LiOH precursor. The presence of hydrogen in the LiOH precursors creates a high background due to incoherent scattering, which persists until the phase reaches its melting point at 485 °C. In region I, prior to the LiOH melting, the reflections of the cubic phase shift subtly to lower angles, indicating an increase in the lattice parameter due to thermal expansion. The reflections later shift to higher angles because of the contraction of the unit cell, resulting from Li incorporation into the structure (and concurrent Ni oxidation). The onset of region II is observed to coincide roughly with the onset of LiOH melting, which is accompanied by further shift in the reflections of the cubic phase to higher angles, indicating a contraction of the lattice parameter. The melting of the LiOH improves the kinetics of the reaction, which would result in a rapid uptake of Li into the now significantly li­thia­ted Li*_y_* Ni_1−*y*_O phase. In region II, the Li and increasingly oxidized Ni in the material begin to form a more ordered crystal structure, resulting in the reduction of the unit-cell volume. This process continues until the temperature approaches the annealing temperature of 700 °C, before which the initial formation of the layered structure can be identified by the characteristic splitting of particular reflections, such as the 220_C_ reflection into the 108_R_ and 110_R_ reflections, which coincides with the beginning of region III of the reaction mechanism.

**Figure 3:**
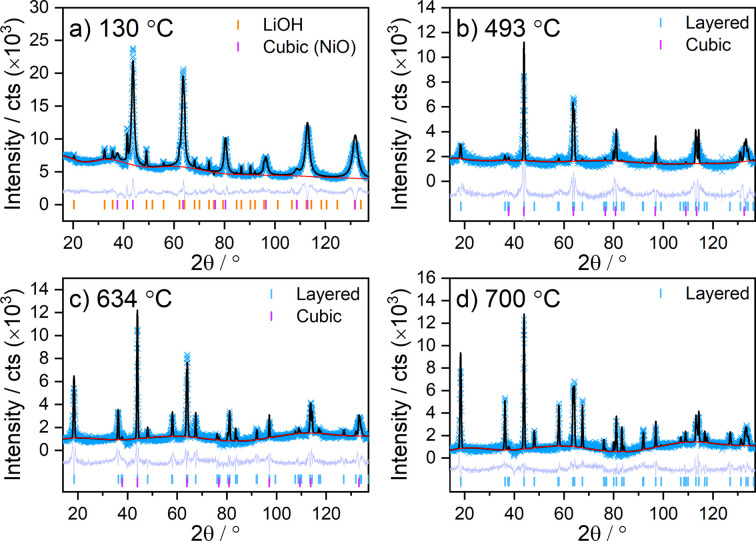
Rietveld refinement profiles and phases identified at various temperatures during the synthesis of LNO: (*a*) 130 °C (*R*_w_ = 12.91%), (*b*) 493 °C (*R*_w_ = 14.31%), (*c*) 634 °C (*R*_w_ = 16.25%) and (*d*) 700 °C (*R*_w_ = 15.92%).

**Figure 4:**
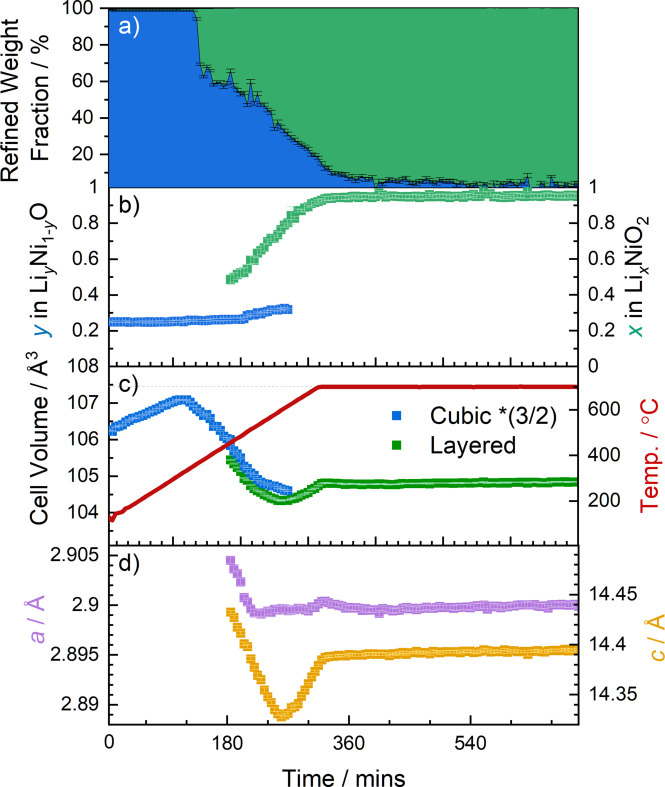
Refined structural parameters of the cubic (blue) and layered (green) phases during the synthesis of LNO. (*a*) Weight fractions, (*b*) Li site occupancies, (*c*) unit-cell volumes and sample temperature (red solid line), and (*d*) *a* and *c* cell parameters of the layered phase.

To identify the phases present and their structural parameters during synthesis more precisely, Rietveld analysis was used to refine structural models against the diffraction patterns collected during the *in situ* experiment. Examples of the quality of fit and the structures identified at various temperatures are shown in Fig. 3 ▸. The refined structural parameters of the cubic and layered phases during the synthesis of LNO are presented in Fig. 4 ▸. Fig. 4 ▸(*a*) shows the weight fraction of each phase determined from the relative scattering intensity observed during the experiment. The cubic phase is the predominant phase in the mixture at the beginning of the experiment and, as discussed earlier, reflections from the layered phase are not observed until the temperature of the sample environment reaches ≈460 °C. The cell volume of the initial cubic phase increases at first, which can be attributed to thermal expansion. However, as the temperature reaches ≈330 °C, it begins to decrease as lithium diffuses into the structure [see Figs. 4 ▸(*b*) and 4 ▸(*c*)]. Although larger Li^+^ ions [*r*(Li^+^) = 0.76 Å] are incorporated into the cubic structure, this is accompanied by the oxidation of the Ni^2+^ host ions to ions with smaller radius [*r*(Ni^2+^) = 0.69 Å, *r*(Ni^3+^) = 0.56 Å)] (Shannon, 1976 ▸), which are distributed over the available octahedral sites. This explains the overall contraction of the cell volume until it reaches a minimum value of 104.60 (2) Å^3^, coinciding with a maximum Li content of *y* = 0.318 (7) in the cubic phase. Further li­thia­tion results in Li and Ni ordering in the structure, and this is accompanied by the emergence of characteristic reflections from the layered phase as discussed above. The layered phase becomes the dominant phase in the mixture at ≈600 °C and exhibits an initial refined Li site occupancy of *x*(Li) ≈ 0.51 [Fig. 4 ▸(*b*)]. The segregation of Li and Ni into separate layers results in further expansion of the structure until the cell parameters stabilize as the sample temperature reaches the annealing temperature of 700 °C. During annealing, the Li site (3*b*) occupancy also stabilizes at a value of *x*(Li) ≈ 0.95, while there is a subtle increase in cell volume. Yet, some amount of Li is also observed in the Ni site (3*a*). Fig. 8(*a*), which is explored in further detail later on, shows the refined site occupancies of Li in the layered phase during annealing and subsequent cooling of the sample environment. As the sample is cooled to ambient temperature, the site occupancies of Li begin to stabilize and converge towards values of 0.981 (6) and 0.019 (6) for the Li (3*b*) and Ni (3*a*) layers, respectively. This could be related either to an actual improved ordering of the sample during cooling or to an improved fitting of the site occupancies due to the lower values of the Debye–Waller factors at lower temperature. Either way, these results suggest that the experimental setup used here is able to produce high-quality LNO with minimal off-stoichiometry or site (point) defects, comparable to synthesis in the laboratory. In contrast, during the previously reported synchrotron study a higher off-stoichiometry was observed at the end of the experiment (Li_0.95_Ni_1.05_O_2_) due to Li loss to reaction with the quartz (Bianchini, Fauth *et al.*, 2020 ▸). The phase evolution observed in both studies is consistent, starting with the gradual li­thia­tion of the rock salt-type phase to form Li*_y_* Ni_1−*y*_O. This li­thia­tion is accelerated as the temperature of the sample environment exceeds the melting point of LiOH, and neutron data suggest that the improved reaction kinetics after melting are still observed despite the larger sample mass. No intermediate rock salt-type phases of specific composition are observed, as was the case with the synchrotron study, suggesting that homogeneous li­thia­tion of the entire sample mass is achieved thanks to even heating of the entire quartz-tube volume. The subsequent ordering of Ni and Li in the structure to form a ‘defective’ LNO phase is first observed at *T* ≈ 436 °C in the synchrotron study; however, with the larger sample mass used here, and possibly due also to the lower resolution of neutron diffraction, the Bragg reflections corresponding to such a ‘defective’ layered LNO phase are not observed until *T* ≈ 460 °C. Subsequent ordering of Ni and Li in the structure enables the LNO to become the dominant phase in the mixture beyond *T* ≈ 600 °C in both cases, suggesting that this behaviour is dependent on thermal activation rather than even distribution of Li/Ni within the mixture. As the mixtures were further heated towards the annealing temperature of 700 °C, further ordering was suggested in the synchrotron study by monitoring the slab and interslab distances of the layered phase; however, as shown in Fig. 7 and discussed later in the text, in this study neutron data enable the distribution of Ni and Li in the layered phase to be resolved directly as a function of temperature. The final stoichiometry is closer to the target one due to the reduced number of side reactions of LiOH with SiO_2_, which in the synchrotron study was observed to form Li_4_SiO_4_.

**Figure 5:**
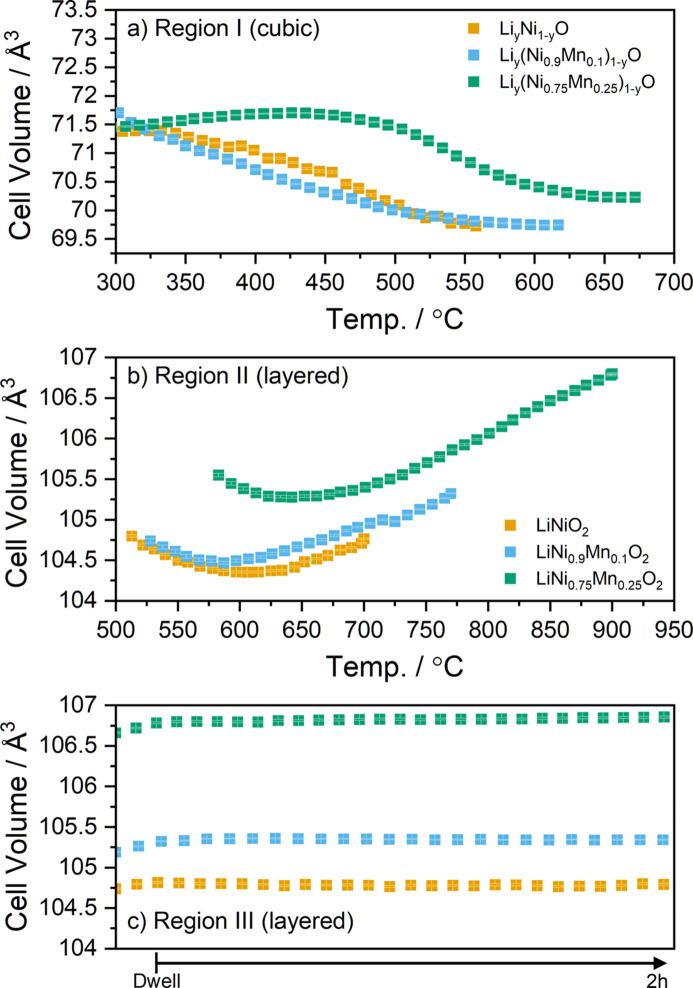
Unit-cell volume of crystalline phases observed during the three regions of the synthesis process. (*a*) Rock salt-type cubic phases, (*b*) layered phases during the temperature ramp and (*c*) layered phases observed during a 2 h dwell at the targeted annealing temperature. Here, the different values of the volumes are also due to the different thermal expansion at the temperatures chosen for annealing.

Having established that this method is suitable to study the synthesis of layered oxide cathode materials, we compare the behaviour of the LNO mixture with two Mn-containing materials (NM9010 and NM7525, with 10 and 25% Mn content, respectively), which were synthesized under similar conditions. Similarly to LNO, these materials are prepared by li­thia­tion of a hydroxide precursor of appropriate stoichiometry (Ni:Mn ratio), which was in this case not pre-annealed. The reaction also proceeds via the formation of a cubic phase, which is li­thia­ted before the subsequent formation of the layered phase. As is commonly reported in the literature, the presence of Mn in the structure necessitates higher annealing temperatures to form a pure layered phase (Liu *et al.*, 2022 ▸; McCalla & Dahn, 2013 ▸). Previous studies have also shown that the reaction pathway and observed intermediate phases differ markedly for various cathode-material compositions despite targeting the same final layered structure (Ying *et al.*, 2023 ▸; Duffiet *et al.*, 2022 ▸; Hua *et al.*, 2019 ▸). Qualitatively, the *in situ* diffraction data collected from the Mn-containing mixtures look very similar to the LNO data shown earlier in Fig. 2 ▸, and are gathered in Fig. S2. To compare the behaviour of the different systems, we carried out analogous Rietveld refinement of the *in situ* diffraction data obtained in the three cases. Fig. 5 ▸ shows the evolution of the unit-cell volumes of each phase as a function of temperature. The cell volumes as a function of time are shown in Fig. S3. As may immediately be seen in region I [Fig. 5 ▸(*a*)], while a small amount of Mn (NM9010) has little effect on the evolution of cell volume versus temperature, the NM7525 sample shows a significantly delayed onset for the li­thia­tion process. In fact, thermal expansion (cell volume increase) dominates over li­thia­tion (cell volume decrease) until 450 °C, while this value was close to 300 °C for the other two samples. Hence, it is not surprising that the rock salt phase is observed up to higher temperatures of 675 °C for the NM7525 sample, *i.e.* the li­thia­tion of the rock salt-type phase requires more time and higher temperature.

**Figure 6:**
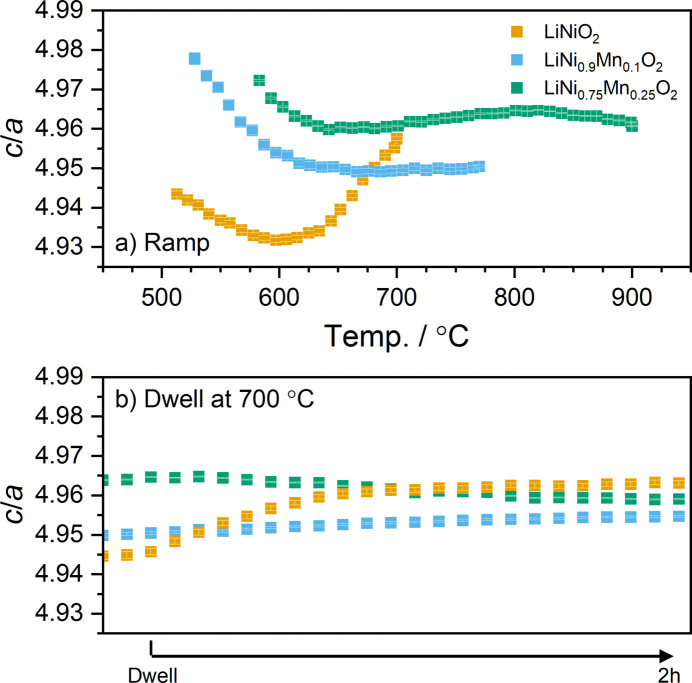
Observed *c*/*a* ratio of the layered phase during (*a*) the temperature ramp and (*b*) the 2 h dwell at the targeted annealing temperature. The dwell occurs at different temperatures for the three samples.

In region II [Fig. 5 ▸(*b*)], again the behaviour of the cell volume for the LNO and NM9010 samples is rather close, while NM7525 shows a very delayed response to the heating process, with a significant volume increase observed all the way to 900 °C. In all three cases, during the dwell [Fig. 5 ▸(*c*)] there is little change of unit-cell volume, with the three samples stabilizing rapidly to their asymptotic values. Here again, the difference between LNO and NM9010 is much less pronounced than that between NM9010 and NM7525, indicating a nonlinear behaviour of the samples with increasing Mn content. In other words, an increasing Mn content results in a more than linear increase in the delay in the li­thia­tion process, as well as in the final cell volume. These observations also explain the requirement for the annealing temperature to rise more than linearly (*i.e.* 700, 770 and 900 °C for increasing Mn content). This behaviour is reflected in the evolution of the *c*/*a* ratio during annealing, presented in Fig. 6 ▸. In all mixtures, it decreases to a minimum value, and the temperature at which this minimum is observed increases as the Mn content of the material is increased. The increase in the *c*/*a* ratio beyond this point is indicative of the onset of Li and TM ordering in the structure, which causes a rhombohedral distortion of the rock salt structure. During the dwell, the *c*/*a* ratio of the three materials appears to stabilize initially; however, it is then found to slowly decrease for NM7525. The higher annealing temperature required for this sample may result in higher lithium evaporation, which induces greater Li:Ni intermixing, causing the observed reduction in the *c*/*a* ratio. This is confirmed in the later discussion of site occupancies, where NM7525 was found to have the highest concentration of Li in the Ni layer (3*a* site) after cooling (see Table 1 ▸). This highlights the necessity to increase the Li content in the precursor mixture when preparing Ni-rich materials at higher temperatures (Langdon & Manthiram, 2021 ▸).

**Figure 7:**
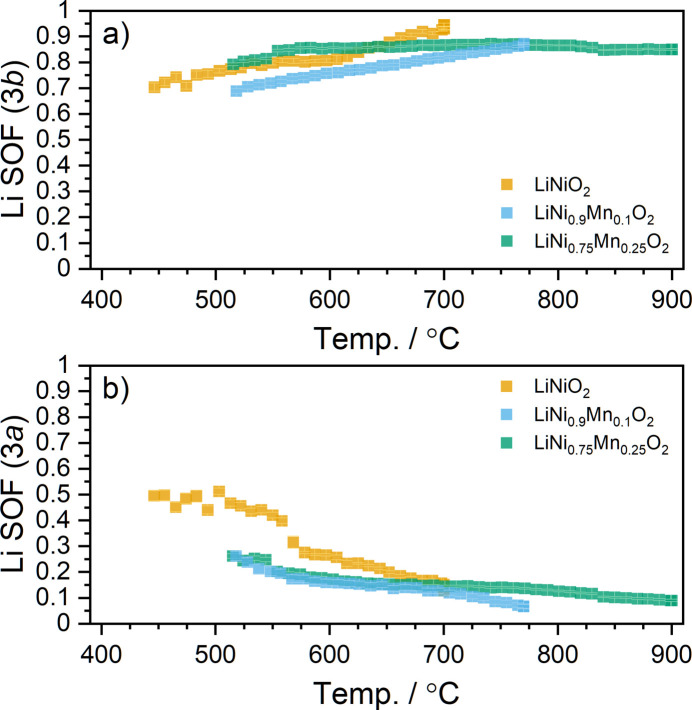
Refined SOFs of Li as a function of temperature upon heating. (*a*) Li in the Li layer (3*b* site) and (*b*) Li in the TM layer (3*a* site).

**Figure 8:**
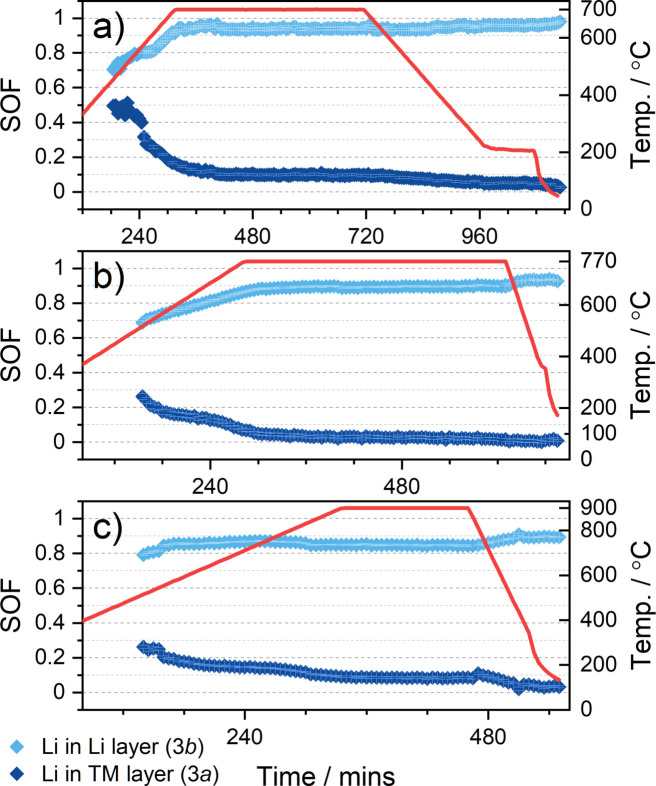
Refined SOFs of Li in the 3*b* site (Li layer, light blue) and 3*a* site (TM layer, dark blue) in the layered structure during annealing and cooling of the three mixtures. (*a*) LNO (*b*) NM9010 and (*c*) NM7525.

The refined SOFs of Li in the 3*a* and 3*b* sites of the layered structures during the annealing and cooling of the mixtures are shown in Figs. 7 ▸ and 8 ▸. Some constraints were applied in the refinement of structural models to reflect the expected chemistry of the materials during synthesis. The Mn content of the 3*a* site was fixed to 0.25, 0.1 or 0 as appropriate, while the total occupancy of Ni on the 3*a* and 3*b* sites was constrained to 0.75, 0.9 or 1 accordingly. As the materials are undergoing li­thia­tion during data collection, it cannot be assumed that the layered structures achieve stoichiometric li­thia­tion, and thus the total Li occupancy across the 3*a* and 3*b* sites was not constrained. The Mn content is fixed, while the total Ni amount is fixed but its proportion in the 3*a* and 3*b* sites is allowed to vary. The presence of Mn^4+^ in the structure means that at any given time the residual amount of Ni^2+^ is higher than in the Mn-free case, which increases the amount of off-stoichiometry. Thus the materials with higher Mn content are observed to have a lower Li site occupancy in the 3*b* site, especially beyond 650 °C. The Mn-containing materials are also found to have a significantly larger cell volume and *c*/*a* ratio (see Table 1 ▸). Despite the smaller ionic radius of Mn^4+^, this trend can be attributed to both an increase in off-stoichiometry and a higher concentration of Ni^2+^, which forms due to charge balance with the substituted Mn^4+^. Fig. 7 ▸ shows how the refined Li site occupancies evolve as a function of temperature, and here it is evident that increasing the Mn content in the material reduces the time required for ordering of Li and Ni in the layered structure. Although this may seem counterintuitive, it may be explained by the larger unit-cell size of both the rock salt-type and layered structure in the presence of a large amount of Mn. The larger cell has larger diffusion channels for Li, which can hence more quickly order on the 3*b* sites. In the 25% Mn mixture, the Li_3*b*_ occupancy stabilizes at a value of ≈0.87 ± 0.01 before the mixture reaches the targeted annealing temperature of 900 °C. In contrast, the LNO and NM9010 mixtures have not yet been completely li­thia­ted by the time they reach their annealing temperatures of 700 and 770 °C, respectively, and the Li site occupancies continue to evolve during the dwell as shown in Fig. 8 ▸. In summary, our data demonstrate that while the rock salt-type phase endures longer in Mn-containing samples, resulting in a longer required reaction time and higher calcination temperature, the Li occupancy in the 3*b* site also increases more quickly, indicating faster Li diffusion in these materials. The differing evolution of the different structural parameters discussed above clearly highlights the delay in the onset of the li­thia­tion as the composition is varied to include higher Mn content at the expense of Ni. Finally, as shown in Table 1 ▸, we also find that the product materials are not fully li­thia­ted: while the total Li content in LNO is observed to be as close to 1 as expected, this decreases as the Mn content increases. We find 0.95 (1) and 0.93 (1) for LiNi_0.9_Mn_0.1_O_2_ and LiNi_0.75_Mn_0.25_O_2_, respectively (which then would actually be written more appropriately as Li_0.95_Ni_0.9_Mn_0.1_O_2_ and Li_0.93_Ni_0.75_Mn_0.25_O_2_). This implies that the higher calcination temperature results in more Li loss and therefore under-li­thia­ted samples, which may be expected to negatively affect the capacity of such materials.

## 4. Conclusions

The synthesis of three materials, LiNiO_2_, LiNi_0.9_Mn_0.1_O_2_ and LiNi_0.75_Mn_0.25_O_2_, has been resolved in real time by collecting neutron diffraction data *in situ* while heating mixtures of hydroxide precursors in a variable-temperature sample environment. Rietveld refinement was further used to model the structural evolution taking place during the heating and subsequent cooling of the mixtures. As previously reported, the hydroxide precursors firstly dehydrate to form rock salt-type oxides, and the li­thia­tion of these oxides is found to occur in distinct steps as a function of temperature. In this study, the li­thia­tion process was shown to take place in three regions: beginning with (I) the gradual li­thia­tion of the cubic rock salt phase, then (II) the diffusion and gradual ordering of Li^+^ ions within the cubic structure to form a defective layered structure, and finally (III) the formation of a layered oxide material with long-range separation of Li and Ni/Mn in distinct layers. The comparison of the three materials with varying Mn content studied here demonstrates that the onset of the li­thia­tion and subsequent Li/TM ordering requires higher temperature as the Mn content in the material is increased. However, the higher required temperatures also seem to result in some Li loss in the Mn-containing samples, *i.e.* the products are not fully li­thia­ted in these cases. This study validates the effectiveness of neutron diffraction for investigating solid-state synthesis reactions in real time, with material quantities analogous to what is used for small-scale pilot production in industry. The final products were also of the desired stoichiometry, with Li loss congruous to what is typically observed in a furnace. The findings presented here further validate previous studies of the synthesis of LiNiO_2_, while the comparison with other Ni-rich materials, LiNi_0.9_Mn_0.1_O_2_ and LiNi_0.75_Mn_0.25_O_2_, demonstrates the difference in li­thia­tion behaviour with varying Ni content in the structure. A higher annealing temperature is necessary to achieve perfect layering as Ni is substituted with Mn, as the rock salt phase persists at higher temperature. However, the use of higher annealing temperatures and longer annealing times also promotes greater Li loss, which was reflected in the lower Li stoichiometry of the Mn-substituted materials synthesized in this study. These findings hence provide insight into the dynamic behaviour of the reactants and products in the synthesis of Ni-rich cathode materials, and provide direction to better optimize parameters such as the annealing time and temperature in the industrial production of these materials.

## Supplementary Material

Supporting information. DOI: 10.1107/S1600576723004909/jo5081sup1.pdf

## Figures and Tables

**Table 1:** Refined structural parameters of the synthesized materials after cooling to 200 °C (and at ambient temperature for LNO) Corresponding Rietveld refinement profiles are shown in Fig. S4.

Sample	*R*_w_ (%)	*a* (Å)	*c* (Å)	*V* (Å^3^)	*c*/*a*	Li in Li layer (3*b*)	Li in TM layer (3*a*)	Total Li
LiNiO_2_ (47 °C)	20.97	2.8737 (2)	14.1831 (9)	101.432 (8)	4.936 (4)	0.981 (6)	0.019 (6)	1.000 (8)
LiNiO_2_ (200 °C)	20.22	2.8780 (2)	14.2168 (9)	101.981 (8)	4.940 (4)	0.961 (5)	0.047 (5)	1.008 (7)
LiNi_0.9_Mn_0.1_O_2_	8.02	2.8828 (1)	14.2479 (4)	102.545 (4)	4.942 (2)	0.934 (4)	0.019 (7)	0.953 (8)
LiNi_0.75_Mn_0.25_O_2_	11.31	2.8870 (2)	14.2790 (9)	103.069 (8)	4.946 (4)	0.898 (4)	0.028 (7)	0.926 (8)
