# Supplementary material for: In situ neutron diffraction to investigate the solid-state synthesis of Ni-rich cathode materials
Source: J Appl Crystallogr. 2023 Jun 23;56(Pt 4):1066–75. doi: 10.1107/S1600576723004909 (PMC10405595; doi:10.1107/S1600576723004909)
Supplement: Supplementary file 1 [file j-56-01066-sup1.pdf]

JOURNAL OF
APPLIED
CRYSTALLOGRAPHY

Volume 56 (2023)

Supporting information for article:

In situ neutron diffraction to investigate the solid-state synthesis of Ni-rich cathode materials

Damian Goonetilleke, Emmanuelle Suard, Benjamin Bergner, Jürgen Janek, Torsten Brezesinski and Matteo Bianchini

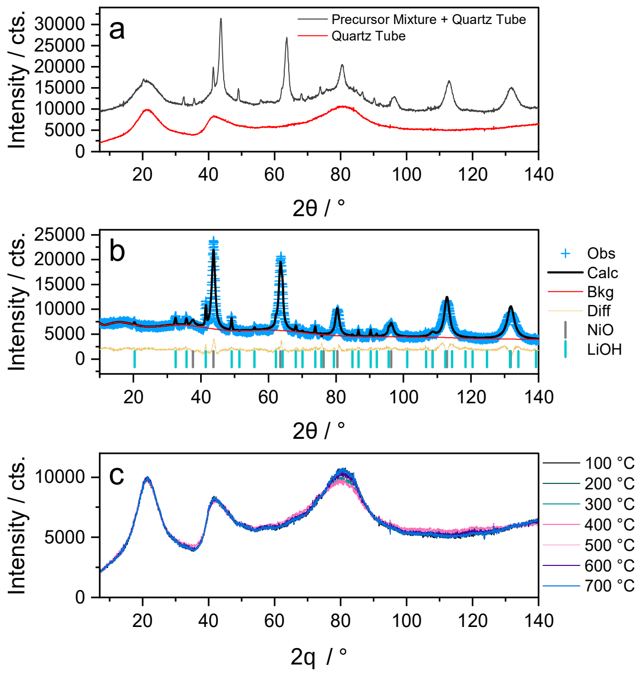

Figure S1 a) Raw data collected from the initial pattern of the LiOH + NiO heating experiment and an empty quartz tube. b) Example of Rietveld refinement profile for the initial pattern of the LiOH + NiO heating experiment after subtraction of the quartz tube background profile. c) Evolution of diffraction patterns collected from an empty quartz tube at various temperatures.

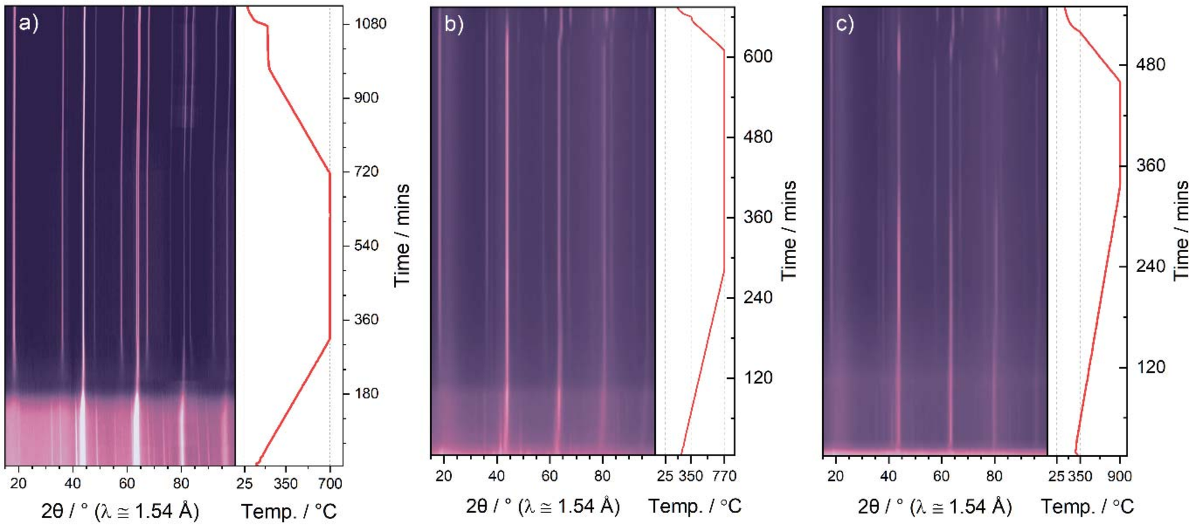

Figure S2 Contour plots of diffraction data collected during heating and then cooling of the three mixtures targeting a) $LiNiO_2$, b) $LiNi_{0.9}Mn_{0.1}O_2$ and c) $LiNi_{0.75}Mn_{0.25}O_2$.

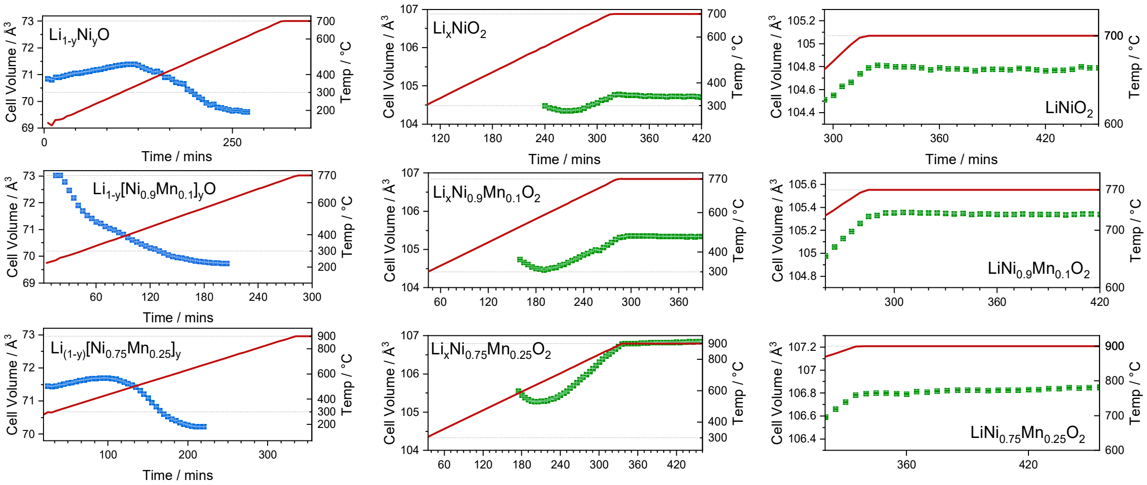

Figure S3 Unit cell volume of the cubic (left) and layered phases (middle, right) as a function of time during the heating experiments.

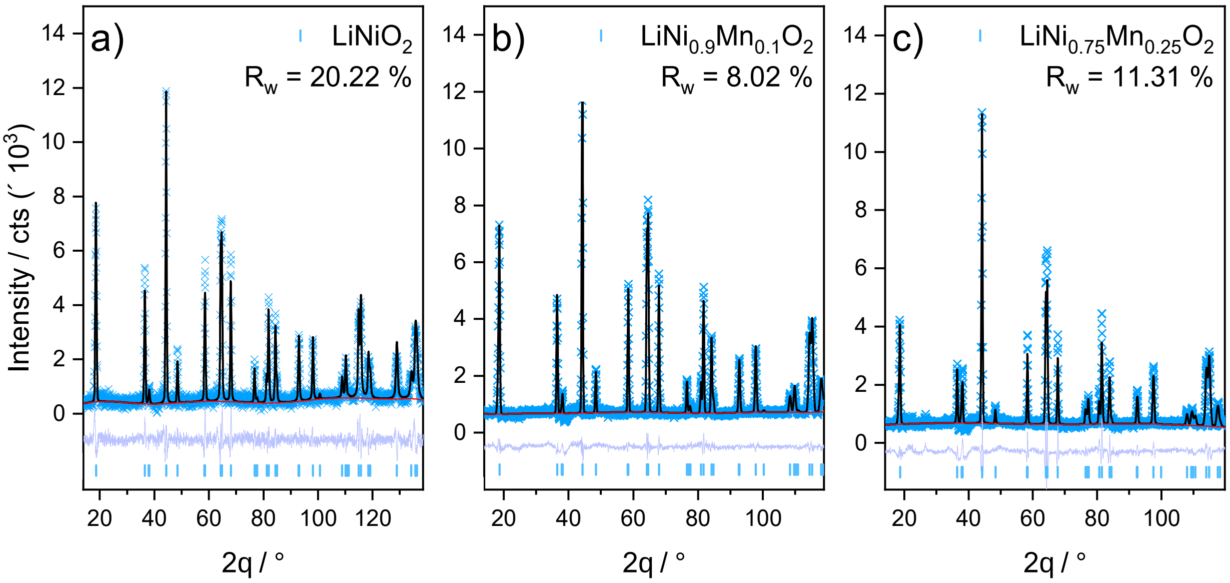

Figure S4 Rietveld refinement profiles of the synthesised materials: a) $LiNiO_2$, b) $LiNi_{0.9}Mn_{0.1}O_2$ and c) $LiNi_{0.75}Mn_{0.25}O_2$ after cooling to 200 °C.

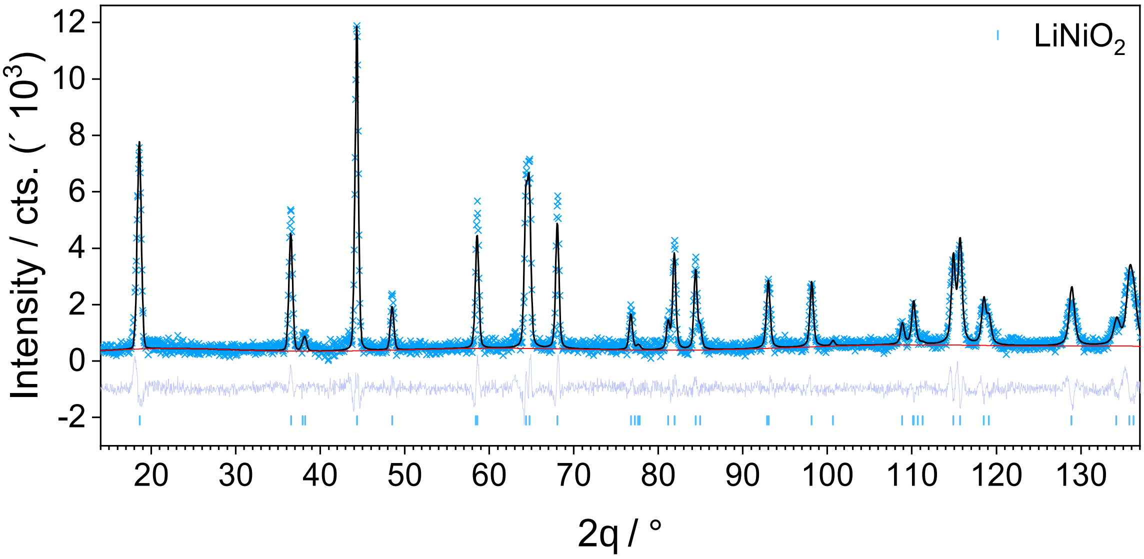

Figure S5 Rietveld refinement profile of the synthesised $LiNiO_2$ phase after cooling to ambient temperature.

Table S1 Structure parameters and atomic co-ordinates of the synthesised $LiNiO_2$ phase after cooling to ambient temperature.

**Atom**	**Site**	**Sym.**	**x**	**y**	**z**	**frac**	**U_{iso} / Å^2**
**Li1**	3b	-3m	0	0	0.5	0.981(6)	0.00165
**Ni1**	3b	-3m	0	0	0.5	0.019(6)	0.00165
**Li2**	3a	-3m	0	0	0	0.019(6)	0.00788
**Ni2**	3a	-3m	0	0	0	0.981(6)	0.00788
**Mn2**	3a	-3m	0	0	0	0.000	0.00788
**O**	6c	3m	0	0	0.25797(27)	1.000	0.00877
**Space group = $R\overline{3}m$, R_w = 20.97 %, R_F = 7.69 % *a* = 2.8737(2) Å, *c* = 14.1831(9) Å, Vol = 101.432(8) Å^3**							
